# Enriched Surface Oxygen Vacancies of Fe_2_(MoO_4_)_3_ Catalysts for a PDS-Activated photoFenton System

**DOI:** 10.3390/molecules28010333

**Published:** 2022-12-31

**Authors:** Yang Qiu, Chuanxi Yang, Huimin Zhou, Jinqiu Zang, Yuqi Fan, Feng Dang, Guanwei Cui, Weiliang Wang

**Affiliations:** 1 Institute of Environment and Ecology, Shandong Normal University, Jinan 250358, China; 2 School of Environmental and Municipal Engineering, Qingdao University of Technology, Qingdao 266520, China; 3 Key Laboratory for Liquid-Solid Structural Evolution and Processing of Materials, Shandong University, Jinan 250061, China; 4 Key Laboratory of Molecular and Nano Probes, College of Chemistry, Chemical Engineering and Materials Science, Ministry of Education, Shandong Normal University, Jinan 250014, China

**Keywords:** Fe_2_(MoO_4_)_3_, photoFenton, shuttle, synergistic effect, oxygen vacancies

## Abstract

The environmentally benign Fe_2_(MoO_4_)_3_ plays a crucial role in the transformation of organic contaminants, either through catalytically decomposing oxidants or through directly oxidizing the target pollutants. Because of their dual roles and the complex surface chemical reactions, the mechanism involved in Fe_2_(MoO_4_)_3_-catalyzed PDS activation processes remains obscure. In this study, Fe_2_(MoO_4_)_3_ was prepared via the hydrothermal and calcine method, and photoFenton degradation of methyl orange (MO) was used to evaluate the catalytic performance of Fe_2_(MoO_4_)_3_. Fe_2_(MoO_4_)_3_ catalysts with abundant surface oxygen vacancies were used to construct a synergistic system involving a photocatalyst and PDS activation. The oxygen vacancies and Fe^2+^/Fe^3+^ shuttle played key roles in the novel pathways for generation of •O_2_^−^, h^+^, and ^1^O_2_ in the UV–Vis + PDS + FMO-6 photoFenton system. This study advances the fundamental understanding of the underlying mechanism involved in the transition metal oxide-catalyzed PDS activation processes.

## 1. Introduction

Advanced oxidation processes (AOPs) are considered to be effective methods for degrading organic pollutants [1,2]. Fenton technology has been widely applied in wastewater treatment [3]. However, the widespread use of Fenton technology is limited by pH [4]. Consequently, advanced oxidation with sulfate radicals has attracted much attention. This is because persulfate ($S_{2}O_{8}^{2-}$) (E_0_ = 2.01 V) has a higher potential than H_2_O_2_ (E_0_ = 1.76 V), and the reactivity is not affected by pH [5,6]. In general, persulfates (PS) can be activated by light, heat, microwaves, and some transition metals and their oxides. Transition metals are considered promising catalysts for application in advanced oxidation systems with sulfate radicals due to the presence of empty orbitals and variable valence states [7,8,9,10,11,12]. Among them is the Fe^3+^/Fe^2+^ electron shuttle system involving iron compounds, which effectively activate PS. Therefore, finding a suitable iron-based catalyst is the key to building an AOPS system.

Iron molybdate, Fe_2_(MoO_4_)_3_, is a new photocatalytic material that has the advantages of other molybdates, but its unique crystal structure makes it particularly unusual due to crystal defects. The garnet-like structure of iron molybdate does not have A-site ions occupying the dodecahedral sites in the garnet structure, and each tetrahedron and octahedron are connected at the same top, offering extremely high electron conduction, a stable chemical structure, and great scope for morphological modulation [13,14]. However, Fe_2_(MoO_4_)_3_ exhibits fast electron-hole recommendation, slow carrier migration, and a low visible light absorption efficiency, all of which greatly hinder broad application of Fe_2_(MoO_4_)_3_ photocatalysts [15,16]. Studies have shown that there is synergy between photoactivated PS and photocatalysis, and that the construction of a photoFenton system with UV–vis+PDS+FMO to increase the rate of production of reactive oxygen species is an effective means of improving catalytic activity and pollutant degradation and removal.

The generation pathways for key reactive oxygen species used in oxidative degradation of pollutants remain subjects for debate. Recently, several researchers have reported that nonradical pathways dominate pollutant degradation pathways and free radicals play only minor roles [17,18]. However, Fe_2_(MoO_4_)_3_ has abundant oxygen vacancies that can act as electron transfer centres and mediate electron transfer from the organic substrate to PDS while acting as a catalyst and oxidizer for the Fe^3+^/Fe^2+^ redox cycle. Therefore, we speculated that surface oxygen vacancies and Fe^2+^ should play important roles in the degradation of organic matter via reactions of the photoFenton system [19,20]. It is of interest to further investigate the mechanism of the photoFenton reaction based on Fe_2_(MoO_4_)_3_.

## 2. Results and Discussion

### 2.1. Characterization of FMO

**Figure 1:**
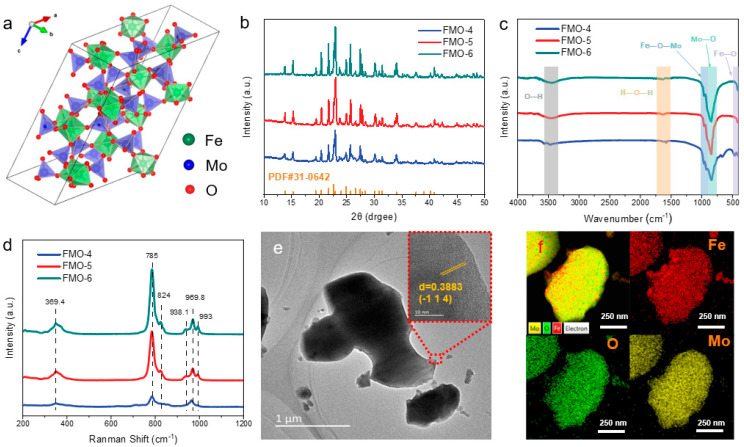
(**a**) Crystal unit cell illustration (blue represents Mo, yellow represents Fe and magenta represents O); (**b**) XRD patterns of the FMO samples; (**c**) FT-IR spectra; (**d**) Raman profiles; (**e**) TEM and HR-TEM images of FMO-6; and (**f**) element maps.

Fe_2_(MoO_4_)_3_ has a unique crystal structure (Figure 1a) with the four top corners of each tetrahedron connected to the male top of the octahedron and the individual tetrahedra separated from each other as well as from individual octahedra. From the powder X-ray diffraction (XRD) plots shown in Figure 1b, the diffraction peaks were indexed as those of Fe_2_(MoO_4_)_3_ (PDF# 31-0642), and all samples were free of any impurity phases. In addition, the diffraction peaks became narrower and more intense as the calcination temperature was increased, which indicated an increase in the crystallinity and particle sizes of FMO, which also brought about structural nuances. This was confirmed by the FT-IR spectrum (Figure 1c), in which vibrational bands in the region 400 to 450 cm^−1^ were attributable to the FeO_6_ octahedra, and the vibrational bands between 700 and 900 cm^−1^ were stretching vibrations of the Mo-O bonds at nonequivalent tetrahedral positions. The weak and narrow bands at 960 to 990 cm^−1^ were assigned to Fe-O-Mo and MoO_4_ [21]. The characteristic peaks at 3440 and 1622 cm^−1^ represented ν(O-H) stretching vibrations and the ν(H-O-H) bending mode, respectively, which are reported to interact with the holes generated during photodegradation [22]. The Raman spectrum (Figure 1d) further revealed the crystal structure of FMO and showed six representative bands for MoO_4_ tetrahedra. The peak at 969.8 cm^−1^ was for symmetric extensions of MoO_4_ tetrahedra, the peak at 785 cm^−1^ was an asymmetric stretching mode, and that at 369.4 cm^−1^ was assigned to a bending mode [23,24].

Figure 1e show typical SEM and TEM images of FMO-6. The catalyst exhibited a sheet structure. The FMO-6 nanosheets were further investigated with high-resolution TEM (HR-TEM). As shown in Figure 1e, the images clearly show a lattice spacing of 0.388 nm corresponding to the (-1 1 4) planes of the Fe_2_(MoO_4_)_3_ crystalline structure. This is in agreement with the selected area electron diffraction (SAED) image (Figure S2). The elemental composition of FMO-6 was determined by X-ray energy dispersive spectroscopy (EDS), as shown in Figure 1f. The atomic ratio of Fe/Mo was approximately 1:2.

**Figure 2:**
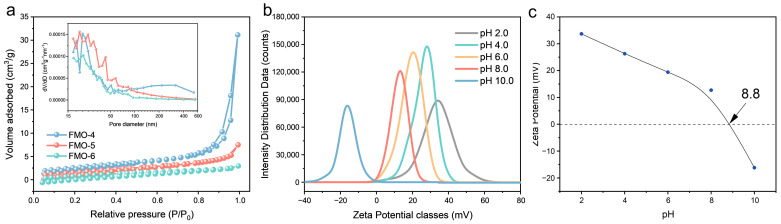
(**a**) Nitrogen adsorption–desorption isotherms (inset: pore size distribution curve for FMO); (**b**) Zeta-potential in solutions of various pH with 2, 4, 6, 8, and 10; (**c**) Zeta potentials of FMO-6 at different pH values.

**Figure 3:**
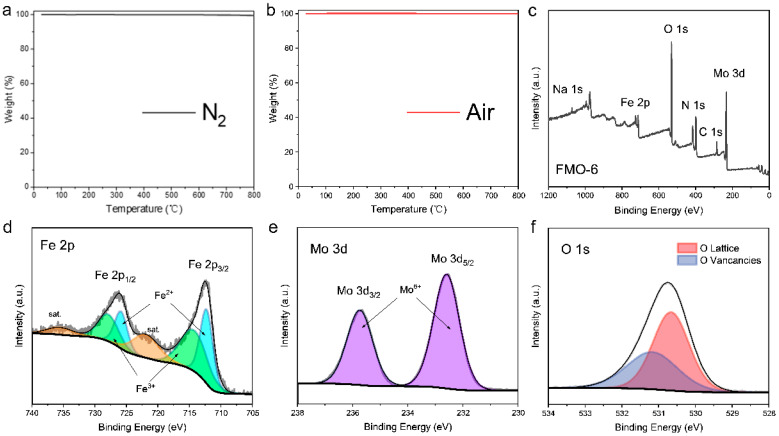
(**a**) TGA analysis of FMO-6 conducted in N_2_; (**b**) TGA analysis of FMO-6 conducted in air; (**c**) full XPS spectrum of FMO-6; high-resolution XPS spectra showing (**d**) Fe 2p, (**e**) Mo 3d, and (**f**) O 1 s binding energies of FMO-6.

The nitrogen adsorption/desorption isotherms for FMO (Figure 2a) indicated a typical type IV mesoporous structure with the H3 hysteresis loop. The BET surface areas of FMO-4, FMO-5 and FMO-6 were 9.40, 6.16, and 4.06 m^2^ g^−1^, respectively, which indicated that the surface areas of FMO decrease with increasing temperature due to expansion of FMO nanoparticle sizes. The inset shows the pore size distribution of the catalyst; as shown in the figure, the pore size increases as the calcination temperature increases from 400 °C to 500 °C, and the pore size distribution decreases when the temperature increases to 600 °C. This may be due to the thermal expansion of the catalyst itself, where the pore size gradually decreases as the catalyst size increases at higher temperatures. The pH-dependent evolution of the zeta potential for FMO-6 is shown in Figure 2b,c. When the pH of the medium was greater than the pH_pzc_ of FMO-6, the catalyst had a negative surface charge; conversely, the surface charge was positive when the pH was lower. It is worth noting, however, that FMO-6 had an extremely high pH_pzc_ of approximately 8.8. To assess the thermal stability of the catalyst, thermogravimetric analyses were carried out by heating from room temperature to 800 °C under air and nitrogen atmospheres (Figure 3a,b). FMO-6 showed only 0.1% weight loss in the air atmosphere and 0.5% in the nitrogen atmosphere, indicating the outstanding stability of the catalyst.

**Figure 4:**
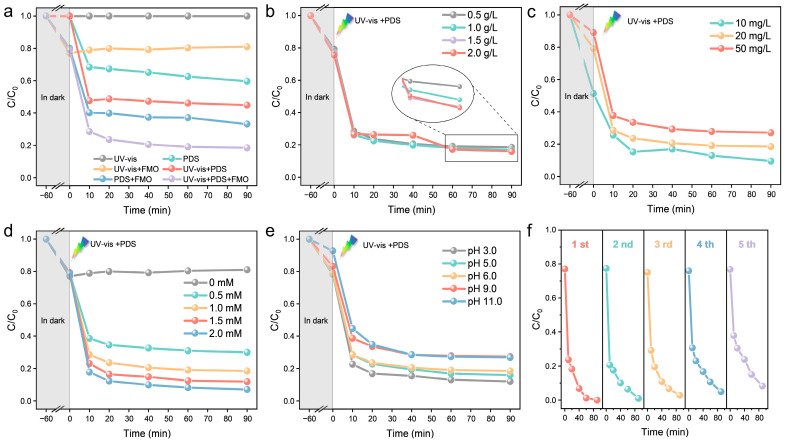
(**a**) Degradation rates of MO under different systems; effects of (**b**) catalyst dosage, (**c**) initial concentration of OFL, (**d**) PMS dosage and (**e**) initial pH; (**f**) reusability of FMO-6.

The valence states of elements on the catalyst surface are strongly correlated with catalyst activity. Therefore, the elemental valence states on the catalyst surface were investigated by X-ray photoelectron spectroscopy (XPS). As shown by the full XPS spectrum in Figure 4c, all elemental components (C, Fe, Mo, and O) were consistent with the chemical structure. The Fe/Mo ratio of FMO-6 was calculated to be approximately 3:5. High-resolution Fe 2p XPS data (Figure 3d) showed that spin-orbit coupling split the Fe 2p peaks into two main features. The binding energies at 712.3 eV and 725.9 eV were for 2p_1/2_ and 2p_3/2_ states, respectively, and the Fe^2+^ oxidation state. The two broader peaks at 722.2 eV and 735.8 eV were attributed to Fe satellite peaks, while the Fe^3+^ binding energies showed peaks at 714.3 eV and 727.9 eV [25,26]. The Mo 3d spectrum (Figure 3e) exhibited two characteristic peaks at 232.6 eV and 235.8 eV, which were assigned to Mo 3d_5/2_ and Mo 3d_3/2_ states, respectively, and the Mo^6+^ oxidation state [27]. Figure 4f shows the O 1 s XPS spectrum with peaks at 530.6 eV and 531.2 eV attributed to lattice oxygen and oxygen vacancies, respectively [28].

### 2.2. Degradation of MO in Different Condition

The catalytic activities of the FMO-4, FMO-5, and FMO-6 catalysts were investigated with three systems, UV–vis, PDS, and UV–vis+PDS. The results showed that FMO-6 exhibited the best catalytic performance (Figure S3). However, the mismatch between the reduced specific surface area and catalytic performance removed the concern that excellent performance differs from a larger contact surface area [29]. All subsequent studies of the photoFenton system used FMO-6 as the catalyst.

**Figure 5:**
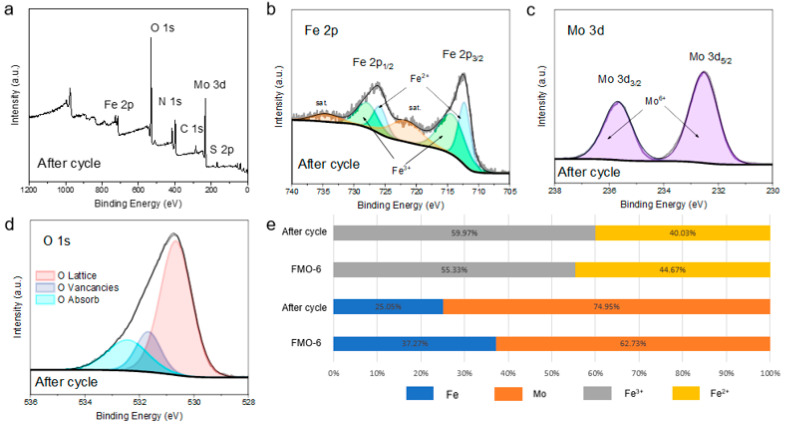
(**a**) the full XPS spectra of FMO-6 after cycling; High-resolution XPS spectra of (**b**) Fe 2p (**c**) Mo 3d and (**d**) O 1 s of FMO-6; (**e**) calculated Fe/Mo ratio and Fe^3+^/Fe^2+^ ratio.

The effects of the FMO-6 system, catalyst dose, pollutant concentration, PDS dose, and pH on the catalytic efficiency were studied by performing five recovery experiments under the same conditions (25 °C, 1 mM PDS, 20 mg/L MO, 0.5 g/L FMO-6, pH = 6, 1000 W Xenon lamp). As shown in Figure 5a, the degradation rates for MO were 0%, 40%, and 55% for the three systems involving UV–vis only, PDS only and UV–vis+PDS, respectively. After the addition of FMO-6, the degradation rates increased to 19%, 66.8%, and 81.5%, respectively. These results showed that FMO-6 had good photocatalytic and PDS activation properties, and there was a synergistic effect between UV–Vis and PDS for the FMO-6 system. Figure 4b shows the effect of catalyst dose on MO degradation efficiency and indicates that there was only a small increase in degradation rate as the dose of FMO-6 was increased. This may be because the FMO-6 catalyst is rich in oxygen vacancies, so a low dose provides enough active sites; therefore, the effect of dose on catalytic activity is not significant. The effect of the initial MO concentration on the degradation efficiency is shown in Figure 4c, which indicates that the degradation efficiency decreased as the pollutant concentration was increased. However, the amount of pollutant removed increased with increasing pollutant concentration, indicating that the catalyst exhibited good catalytic performance. As shown in Figure 4d, there was a significant effect of PDS dose on the degradation rate of MO, which increased with increasing PDS dose. The degradation rate was 70% at 0.5 mM and 93% when the PDS dosage was increased to 2 mM. Figure 5e shows the effect of pH on the rate of degradation for MO. This was consistent with the results in Figure 2c, where there was a significant decrease in the degradation rate of MO when the pH exceeded the pH_PZC_ for FMO-6 (8.8). This may be because at pH 9 and 11, FMO-6 was negatively charged on the surface and repelled $S_{2}O_{8}^{2-}$, thus reducing the efficiency of the system.

Finally, to investigate the reusability of the catalysts, cyclic degradation experiments were carried out under optimal conditions for MO (Figure 4f), and the results showed that the degradation rates were 100%, 99%, 97.25%, 95.09%, and 91.74% for five cycles, which indicated good reusability of the catalysts. The catalysts were characterized by XRD and XPS after cycling, and the XRD results (Figure S4) showed that the structures of the crystals did not change, which confirmed the stabilities of the catalysts. The XPS results showed (Figure 5) that the Mo/Fe ratio changed significantly and the Fe content decreased significantly, which indicates that Fe is the active center of the catalytic reaction. The Fe^2+^ content on the surface of FMO-6 was significantly reduced as compared to that of the initial catalyst, suggesting that the reduced degradation efficiency seen with catalyst reuse was due to blocked conversion of Fe^3+^ to Fe^2+^. Additionally, there were fewer oxygen vacancies after the reaction. The XPS results for Fe 2p and O 1s binding energies before and after the reaction differed. These results indicate that the oxygen vacancies on the catalyst surface are the main active sites and that the electron shuttle cycle of Fe is the key process. The decrease in catalytic activity was due to restricted reduction of Fe^3+^ to Fe^2+^.

### 2.3. Degradation Mechanism by FMO under PDS Activation and Visible Light Irradiation

**Figure 6:**
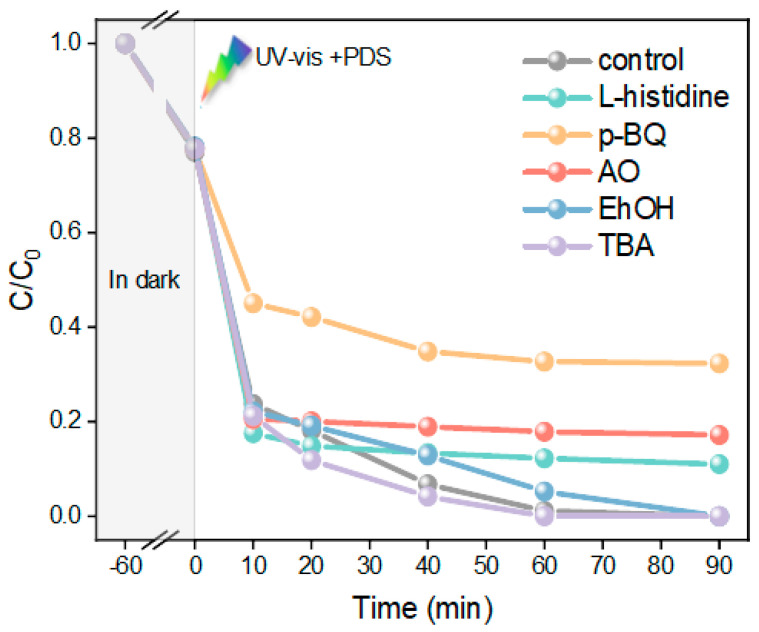
Reactive oxygen species trapping experiments.

To assess the active species in the UV–vis+PDS+FMO-6 system and reveal the reaction mechanism, trapping experiments were carried out, as shown in Figure 6. Here, L-histidine [30], *p*-BQ [31], and AO [32] acted as scavengers of O 12 , $•O_{2}^{-}$ and h^+^. When these trapping agents were added to the system, the MO degradation efficiencies were reduced to 88.98%, 67.64%, and 82.77%, respectively. This indicated that $•O_{2}^{-}$ plays a major role in this system, followed by h^+^ and O 12 . EhOH can be utilized as a scavenger of $SO_{4}^{•-}$ and •OH, and TBA can be used as a scavenger of •OH [33,34]. Surprisingly, when TBA and EhOH were added to the system separately, it was seen that •OH hardly participated in MO degradation, and that $SO_{4}^{•-}$ played a small role.

**Figure 7:**
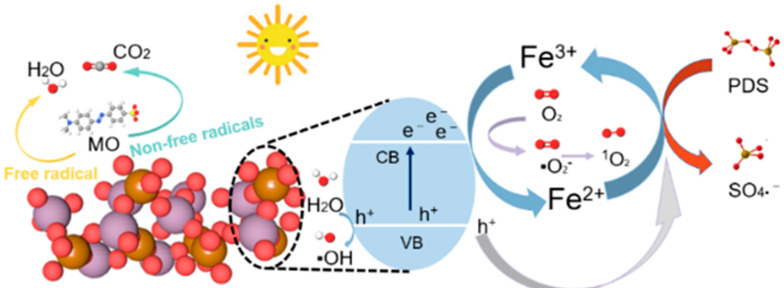
Possible mechanisms for synergistic degradation of MO by the FMO photoFenton system.

Based on these results, we propose a synergistic degradation mechanism (Figure 7) [35,36]. With the use of UV–vis, photogenerated electrons (e^−^) are produced in the conduction band (CB), and photoinduced holes (h^+^) are produced in the valence band (VB) of Fe_2_(MoO_4_)_3_ (Equation (1)) [37]. The difference in carrier transfer rates led to separation of photoinduced e^−^/h^+^ at the interface due to the abundance of oxygen on the surface, which improved the photocatalytic performance. h^+^ can react with the H_2_O in solution to produce •OH (Equation (2)) [38], oxygen in the solution can react with e^−^ to produce $•O_{2}^{-}$ (Equation (3)), and $•O_{2}^{-}$ further reacts with •OH to produce O 12  and OH^−^ (Equation (4)) [39]. Fe^3+^ can also effectively trap e^−^s and directly oxidize organic pollutants, which accelerates the conversion of Fe^3+^ to Fe^2+^ (Equation (5)); upon introduction of PDS, both e^−^ and Fe^2+^ can react with PDS to form large amounts of $SO_{4}^{•-}$ and Fe^3+^ (Equations (6) and (7)) [40,41,42]. The XPS results in Figure 5 also verified that the main active species of FMO-6 was Fe^2+^. Ultimately, MO was degraded to intermediate products and eventually mineralised to CO_2_ and H_2_O, etc., in the presence of $•O_{2}^{-}$, h^+^, O 12  (Equation (8)).

Fe_2_(MoO_4_)_3_ + hv → e^−^ + h^+^ (1)

H_2_O + h^+^ → •OH (2)

(3) $$O_{2}\;+e^{-}\;\to \;•O_{2}^{-}$$

(4) •O2−+•OH → O 12 +OH−

Fe^3+^ + e^−^ → Fe^2+^ (5)

(6) $$S_{2}O_{8}^{2-}+{\mathrm{Fe}}^{2+}\to \;{\mathrm{Fe}}^{3+}+SO_{4}^{•-}$$

(7) $$S_{2}O_{8}^{2-}+e^{-}\;\to \;SO_{4}^{•-}+SO_{4}^{2-}$$

(8) •O2−/h+/O 12  +MO → CO2+H2O+small molecules

## 3. Experiment

### 3.1. Chemicals and Materials

Ammonium persulfate (PDS; 98.5%), iron(III) nitrate nonahydrate (Fe(NO_3_)_3_·9H_2_O; 99.9%), *p*-benzoquinone (*p*-BQ; 99%), *L*-histidine (99.5%), and ammonium oxalate monohydrate (AO; 99.99%) were purchased from Shanghai Macklin Biochemical Co., Ltd. (Shanghai, China). Sodium molybdate dihydrate (Na_2_MoO_4_·2H_2_O; 99%), ammonia solution (NH_4_OH), sulfuric acid (H_2_SO_4_), methanol (MeOH), ethanol (EtOH), tert-butyl alcohol (TBA), and methyl orange (MO) were purchased from Sinopharm Chemical Reagent Co., Ltd. (Shanghai, China). All reagents and chemicals used in this study were of analytical grade and used without further purification.

### 3.2. Preparation of Samples

**Figure 8:**
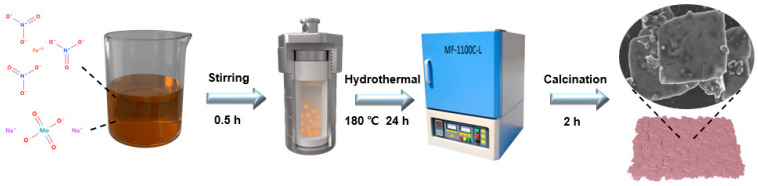
Preparation process for FMO.

Fe_2_(MoO_4_)_3_ was synthesized by a simple hydrothermal method. Briefly, 2.9034 g of Na_2_MoO_4_·2H_2_O and 3.232 g of Fe(NO_3_)_3_·9H_2_O were weighed according to obtain a ratio of Mo/Fe 3:2. Then, the compounds were dissolved separately in 36 mL of deionized water, mixed well, transferred to a hydrothermal reactor, and heated to 180 °C for 24 h. After cooling to room temperature, the resulting precipitate was washed 3 times with ultrapure water and alcohol and dried at 60 °C for 12 h. Finally, they were calcined in an air atmosphere at 2 °C min^−1^ and at different temperatures (400, 500, and 600 °C). The catalyst preparation process is shown in Figure 8.

### 3.3. Characterization

X-ray diffraction (XRD, Rigaku D/Max-r B) was conducted to investigate phase structures with Cu-Kα (λ = 1.540 Å) radiation. The morphologies were examined by field-emission scanning electron microscopy (SEM, Zeiss Gemini 300) and further investigated with high-resolution transmission electron microscopy (HR-TEM, JEM-2100F JEOL LTD, 200 kV). FT-IR spectroscopy (Nicolet IS5) detected the presence of and changes in chemical bonds. Raman spectroscopy (Horiba LabRAM HR Evolution Raman spectrometer) was carried out with a Via Reflex instrument with an excitation laser providing radiation at 532 nm. Specific surface areas and pore size distributions were determined with the N_2_ adsorption–desorption methods of Bruner–Emmett–Teller (BET) and Barrett–Joyner–Halenda (BJH) with a Micrometritics (ASAP 2460) apparatus. Additionally, high-resolution X-ray photoelectron spectroscopy (XPS) was carried out with an ESCALAB 250Xi spectrometer with Al Kα excitation at 280.00 eV to detect surface electronic states and element bonding. Thermogravimetric analysis (TGA) was performed on a TG209F3 system with heating from room temperature to 800 °C in air and a N_2_ atmosphere using a ramp rate of 10 °C min^−1^. The surface potentials of the samples at different solution pH values were obtained with a Nano-Z Zeta potential tester (Malvern Zetasizer Nano ZS).

### 3.4. Catalytic Activation Experiments

In the present work, all degradation experiments were carried out at room temperature (25 ± 2 °C). Batch experiments were carried out in a series of 100 mL beakers containing different concentrations of MO solution using a 1000 W xenon lamp as the light source and 0.05 M H_2_SO_4_ and NH_4_OH to adjust the initial pH. Predetermined amounts of Fe_2_(MoO_4_)_3_ catalyst were added to the beakers, and they were left in the dark for 1 h to reach adsorption equilibrium. After this time, reactions were initiated by adding an appropriate amount of PDS. Aliquots of 3 mL were taken at predetermined time intervals, and an equal volume of MeOH was immediately added to stop the reaction. The mixed solution was filtered through a 0.22 μm membrane filter. Finally, the samples were transferred to a cuvette, and MO concentrations were analysed by ultraviolet–visible (UV–vis) spectrophotometry.

## 4. Conclusions

In summary, Fe_2_(MoO_4_)_3_, which has abundant surface oxygen vacancies, was used as a catalyst to construct a photoFenton system for the degradation of MO. Notably, a new pathway for free radical degradation was generated. This may be attributed to the efficient Fe^3+^/Fe^2+^ redox cycle and its simultaneous use as an active site for photocatalysis and PDS activation in the synergistic system. The present work therefore provides new ideas for interpretation of catalytic processes using transition metal oxides.

## Data Availability

Samples of the compounds are not available from the authors.

## Supplementary Materials

The following supporting information can be downloaded at: https://www.mdpi.com/article/10.3390/molecules28010333/s1, Figure S1: SEM image of FMO-6; Figure S2: SAED image of FMO-6; Figure S3: Catalytic degradation efficiencies for different systems; Figure S4: XRD pattern for FMO-6 after cycling.
