# Supplementary material for: Enriched Surface Oxygen Vacancies of Fe2(MoO4)3 Catalysts for a PDS-Activated photoFenton System
Source: Molecules. 2022 Dec 31;28(1):333. doi: 10.3390/molecules28010333 (PMC9821920; doi:10.3390/molecules28010333)
Supplement: Supplementary file 1 [file molecules-28-00333-s001.zip › molecules-2131871-supplementary.pdf]

Enriched surface oxygen vacancies of $Fe_2(MoO_4)_3$ catalysts for a PDS-activated photoFenton system

Yang Qiu,[a‡] Chuanxi Yang,[b‡] Huimin Zhou,[a] Jinqiu Zang,[b] Yuqi Fan,[a] Feng Dang,[c] Guanwei Cui[d*] and Weiliang Wang[b*]

[a]Institute of Environment and Ecology, Shandong Normal University, Jinan, 250358 China

[b]School of Environmental and Municipal Engineering, Qingdao University of Technology, Qingdao 266520, China

[c]Key Laboratory for Liquid-Solid Structural Evolution and Processing of Materials, Shandong University, Jinan 250061, China

[d]College of Chemistry, Chemical Engineering and Materials Science, Key Laboratory of Molecular and Nano Probes, Ministry of Education, Shandong Normal University, Jinan 250014, China

‡ These authors contribute to this work equally.

* Corresponding authors: cuiguanwei@sdnu.edu.cn (G.W. Cui); sdqcsdnu@163.com (W.L. Wang)

Table of contents

Figure S1. SEM image of FMO-6 3

Figure S2. SAED image of FMO-6 4

Figure S3. Catalytic degradation efficiencies for different systems. 5

Figure S4. XRD pattern for FMO-6 after cycling 6

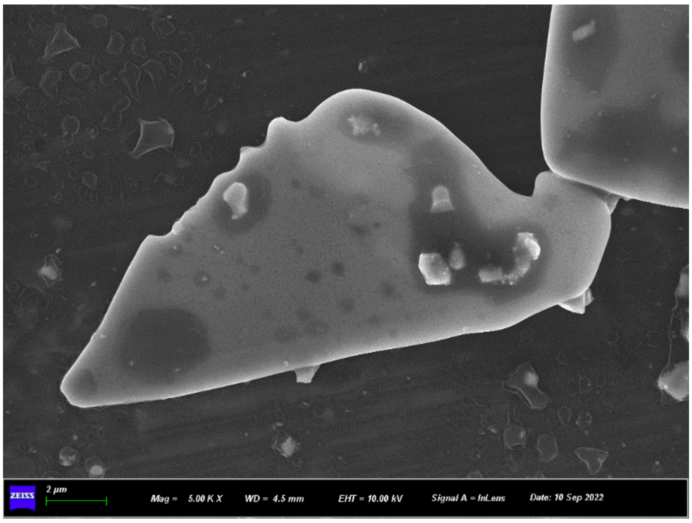

Figure S1. SEM image of FMO-6.

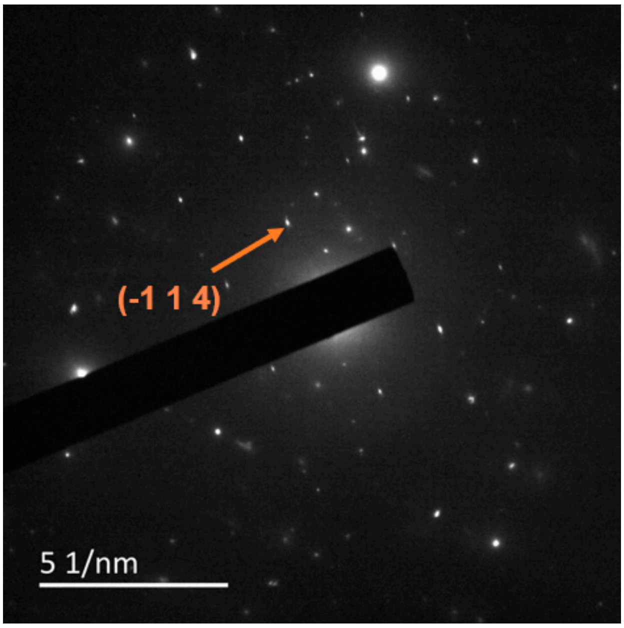

Figure S2. SAED image of FMO-6.

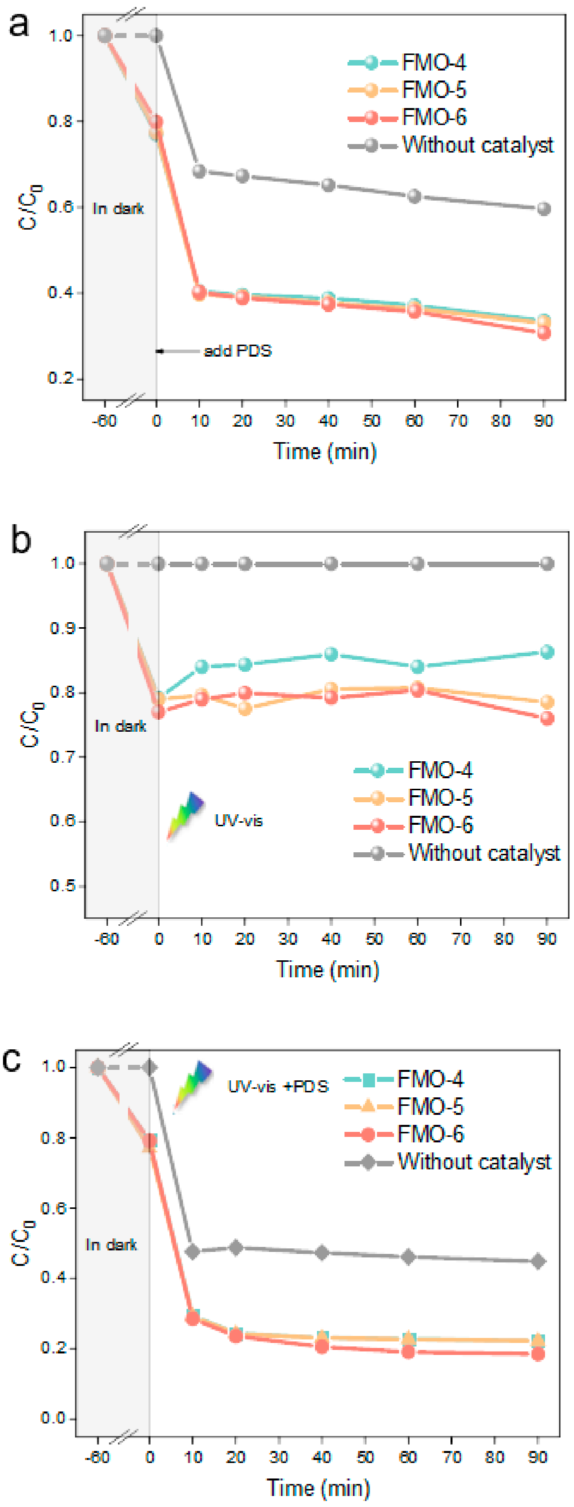

Figure S3. Catalytic degradation efficiencies for different systems (a) PDS, (b) UV-vis, (c) PDS and UV-vis (25℃, 0.5mM PDS, 20mg/L MO, 0.5g/L FMO pH=6, Xenon lamps 1000w).

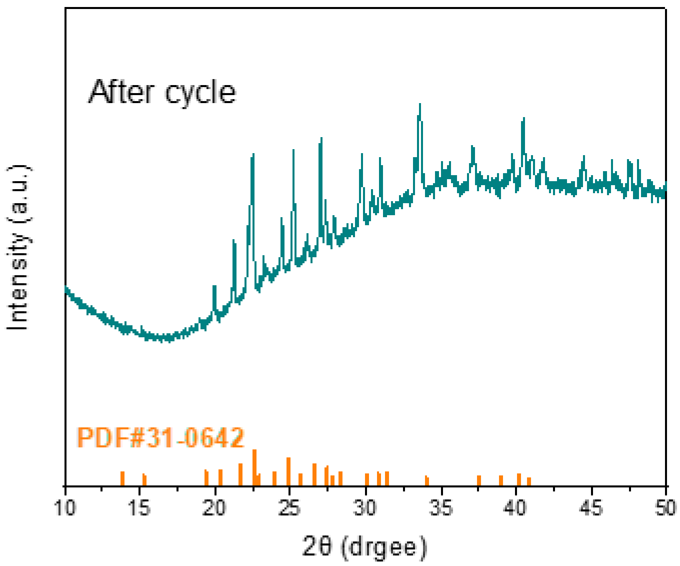

Figure S4. XRD pattern for FMO-6 after cycling.
